# Aneuploidy: An Opportunity Within Single-Cell RNA Sequencing Analysis

**Published:** 2021

**Authors:** Joe R. DELANEY

**Affiliations:** Department of Biochemistry and Molecular Biology, Medical University of South Carolina, Charleston, 29425, USA

**Keywords:** scRNA-seq, Copy-number alterations, Aneuploidy, Cancer

## Abstract

Single-cell sequencing data has transformed the understanding of biological heterogeneity. While many flavors of single-cell sequencing have been developed, single-cell RNA sequencing (scRNA-seq) is currently the most prolific form in published literature. Bioinformatic analysis of differential biology within the population of cells studied relies on inferences and grouping of cells due to the spotty nature of data within individual cell scRNA-seq gene counts. One biologically relevant variable is readily inferred from scRNA-seq gene count tables regardless of individual gene representation within single cells: aneuploidy. Since hundreds of genes are present on chromosome arms, high-quality inferences of aneuploidy can be made from scRNA-seq datasets. This viewpoint summarizes how utilization of these bioinformatic pipelines can benefit scRNA-seq studies, particularly in oncology wherein aneuploidy is both rampant and a hallmark of the studied disease. Awareness and use of these analytical pipelines will improve each field’s ability to understand the studied diseases. Authors are encouraged to attempt these aneuploid analyses when reporting scRNA-seq data, much like copy-number variants are commonly reported in bulk genome sequencing data.

## Introduction

Single-cell RNA sequencing (scRNA-seq) has quickly become a new normal for molecular biology studies, particularly those performed *in vivo*. As cell isolation technology improves, including the advent of spatial partitioning, its use continues to spread. The value is in recognizing cellular heterogeneity within the sample. In oncology, immune cell types can be quickly identified alongside stromal and endothelial cells within the tumor. In neuroscience, glial cells, neurons, astrocytes, oligodendrocytes, and recently differentiated cells can be identified. The Human Cell Atlas seeks to define cellular subtypes in all major organs (Regev *et al*., 2017).

Somatic mutations are now recognized to contribute to clonal heterogeneity within otherwise normal, aged tissue. This recognition comes as ultra-deep sequencing technologies coupled with error-reducing sequencing techniques have enabled the detection of mutant cells occupying less than one percent of an examined tissue sample (Martincorena, 2019). Aneuploidy, the loss or gain of chromosome arms, appears less prevalent in normal tissue in these clonal mutant next-generation sequencing analyses. However, fluorescence *in situ* hybridization studies coupled with specialized single-cell sequencing methods have both highlighted the relatively frequent occurrence of aneuploidy in normal and diseased tissue (Andriani *et al*., 2019). Aneuploidy is known to confer cellular biology effects both dependent and independent of the particular chromosomes altered. The most commonly reported phenotype is the activation of senescence programs in association with aneuploidy.

Given that aneuploidy is (a) known to contribute to cell biology and (b) is present at some detectable level in many cell types, it is valuable for single-cell sequencing studies to include aneuploidy information in their analyses. This is uncommon in the literature. Whereas over 900 tools are available to analyze scRNA-seq data (as monitored on https://www.scrna-tools.org/), only a handful output aneuploidy data or its sub-chromosomal equivalent copy number alterations (CNAs). Yet, reliable aneuploidy calls can be readily obtained in scRNA-seq data; *DNA-sequencing is not required*. In this viewpoint, we direct investigators to select scRNA-seq CNA data analysis tools. We encourage scientists to report stochastic aneuploidy events alongside scRNA-seq datasets.

## Systematic Description

While one might predict that simply mapping read counts per chromosome readily identifies aneuploidy, the reality is that the data are too heterogeneous to quickly determine aneuploidy by eye. Intuitive “eyeball” calls are uncommon in bulk RNA-seq or DNA-seq determination of aneuploidy and CNAs, so it is unsurprising that a simple stacking of read counts is insufficient for quality inference of aneuploidy in scRNA-seq data. Specialized software has thus been developed to appropriately handle RNA-seq data in the context of CNA determination.

Each software package described here recognizes the extreme high noise character of scRNA-seq data. Software may individually benefit from three pieces of information: (1) expression level of many genes along each chromosome arm, (2) changes in B-allele fraction (BAF) including loss of heterozygosity (LOH) in a portion of genes containing sequence variation, or (3) clonality information which restricts noise to a certain level. Aneuploidy is more reliable than focal CNA calls due to the incorporation of data from hundreds of genes. However, smaller CNAs will become more reliable as the depth and read length of RNA sequencing per cell continues to increase with improved capture technologies, sequencing technologies, and decrease of high-throughput sequencing costs.

Peer-reviewed software packages readily infer copy-number alterations from scRNA-seq data: STARCH, (Elyanow *et al*., 2021), InferCNV [Broad Institute], CaSpER (Serin Harmanci *et al*., 2020), clonealign (Campbell *et al*., 2019), and HoneyBADGER (Fan *et al*., 2018). HoneyBADGER is loosely named after its methods: “Hidden Markov Model integrated Bayesian approach for detecting CNVs and LOHs from single-cell RNA-seq data”. While CNA calls are improved based on inclusion of BAF data, HoneyBADGER requires somewhat onerous cell-level separated BAM files as well as a file of pre-defined single-nucleotide polymorphisms (SNPs). CaSpER utilizes a five-state Hidden Markov Model (HMM) alongside BAF to calculate CNAs and removes false positives. CaSpER can also be used with bulk RNA-seq data and determines BAFs from whole-sample aligned BAM files; no pre-defined SNP file is necessary. Like other tools, CaSpER relies on location binning to better build CNAs from many data points. CaSpER uniquely excels in smaller CNA calls due to an ability to detect small scale-specific altered regions, such as focal amplified *PDGFRA*. A limitation to CaSpER is the need to access large aligned files for bulk or single-cell RNA-seq to generate BAFs. The authors of CaSpER note that their software was designed for full transcripts, but made similar calls as HoneyBADGER with a 3’-end scRNA-seq study.

Clonality can be inferred concurrently with CNAs using STARCH or clonealign. Clonealign utilizes single-cell DNA-seq data gathered in parallel to increase the confidence of scRNA-seq CNA calls. Data are then integrated and clonality is estimated for each cell. Clearly, using clonealign strictly to define CNAs in scRNA-seq is circuitous as DNA data are already measured, however, clonality estimates are greatly improved by using both RNA and DNA methods. STARCH was designed to improve analytical calls using spatial information, which presumes clonal expansion requires clones to be more closely packed spatially. However, spatial data is optional to use the tool. Like clonealign, STARCH is able to assign putative clones to single cells.

A uniquely low-prerequisite tool, InferCNV, deserves special attention. It is capable of CNA calls using only count table data from scRNA-seq projects, provided that the user can download a gene and chromosome position file as well as note which cells are “normal”. While InferCNV has been referred to as a visual tool, it is in fact capable of outputting tabular CNA data per cell. While InferCNV has not been peer reviewed in a standalone publication, the math behind it has been utilized in a number of high-impact peer-reviewed publications (Puram *et al*., 2017). A limitation to InferCNV is that due to lack of BAF data or other corrections, it is prone to an increased rate of false-positive calls.

None of these output CNAs explicitly define when aneuploidy occurs. Thresholds are common in the literature: a starting point of 50 percent of a chromosome arm altered in one direction may be considered “aneuploid” in the context of scRNA-seq based CNA data (Kumar *et al*., 2020). This may be adjusted for each study based on false-positive rates in normal, presumably unaltered, cells.

Considering that these software packages are available at no cost yet remain underutilized suggests ease-of-use for users is a potential bottleneck. A point-and-click tool, web-based or otherwise, is unavailable. However, basic scientists already collaborating with bioinformaticians for scRNA-seq data likely have access to individuals capable of using the software highlighted here, as long as the tool’s existence can be communicated to collaborators.

For scientists who cannot readily access the full sequencing data from scRNA-seq runs due to software limitations or bioinformatic limitations, InferCNV may be considered. All tools presented here require R or Python programming knowledge. Incorporation of these tools into point-and-click user interfaces, such as Galaxy (Afgan *et al*., 2018), may increase utilization rates. A summary of these tools is provided in Tab. 1.

## Discussion

Aneuploidy is often a stochastic process. ScRNA-seq is well-poised to quantify patterns of aneuploidy. While scRNA-seq can simultaneously describe the effects of aneuploidy, it cannot by itself contribute to our understanding of aneuploidy’s contribution to cell biology and disease. As aneuploidy is associated with aging and senescence, regulation of the rate of aneuploidy is of interest to many age-related disease fields as well as basic science.

The field with greatest potential benefit from scRNA-seq aneuploidy may be oncology. Aneuploidy is a hallmark of solid tumors and CNAs originating from aneuploidy alter more genes than canonical single-nucleotide variants or small insertion-deletion mutations. Analysis of aneuploidy contributes to our understanding of what forms of genomic instability are present in tumors (Delaney *et al*., 2020). Bulk tumor average aneuploidy and single-cell aneuploidy alike inform alteration frequency and intra-patient heterogeneity. Selective processes of metastasis and chemotherapy are apparent in clusters built from aneuploidy data (Kumar *et al*., 2020). Clinical therapy success may depend on clonal heterogeneity of targetable CNAs, such as loss of *BRCA1*, *BRCA2*, or *BECN1* (Delaney *et al*., 2017), or amplifications of *MET* or *CDK4/6* (Flaherty *et al*., 2020). For immunology studies, aneuploidy calls may increase confidence or support of uniquely “non-tumor” cells as these cells will have markedly different aneuploidy spectrum and normal diploid copy number for the entire genome.

Each of the programs discussed here are capable of producing CNA calls from RNA versions of single-cell sequencing data. Investigators may also pursue DNA-sequencing versions on single-cell experiments, however, the field will benefit from performing aneuploidy analysis within the wealth of scRNA-seq studies already performed, as well as those planned for future studies. Including aneuploidy calls in scRNA-seq workflows is an opportunity ripe for investigation with minimal additional costs.

## Figures and Tables

**TABLE 1:** Summary of available scRNA-seq CNA tools

Tool (# citations, year published)	Expression Input Data Type	Required Secondary Data	Optional Secondary Data	Modeling	BAF Usage	Output	Usage Difficulty	Example Study Using Tool (PMID#)

**CaSpER (13, 2020)**	Aligned scRNA-seq or bulk RNA-seq	–	–	HMM	Yes	CNAs	Moderate	33558546
**Clonealign (32, 2019)**	Gene count tables	Single-cell DNA-seq gene-CNAs	mpileup allele information	Custom	If used as input	CNAs, clones	High	Comparative studies only
HoneyBADGER (83**, **2018)**	Cell-level separated aligned scRNA-seq files,	Single-nucleotide polymorphisms (SNPs)	–	Bayesian, HMM	Yes	CNAs, LOH	High	31747591
**InferCNV (No pub, 2017)**	Gene count tables	–	–	HMM	None	CNAs	Low to Moderate	33121339
**STARCH (5, 2021)**	Gene count tables	–	Spatial Information Table	Hidden Markov Random Field	None	CNAs, clones	Moderate	Reviews Only

Citations were tabulated from Google Scholar in May 2021.

** Tool was co-released with a scientific story; citations may be citing the scientific portion

## Abbreviations

BAF: B-allele fraction
BAM: Binary Alignment Map (.BAM filetype)
CNA: Copy-number alteration
HMM: Hidden Markov Model
LOH: Loss of heterozygosity
scRNA-seq: Single-cell RNA-seq
SNP: Single-nucleotide polymorphism
